# Oral iron preparations: gastrointestinal adverse events and medication adherence in female patients with iron deficiency anemia

**DOI:** 10.3389/fphar.2026.1727566

**Published:** 2026-01-23

**Authors:** Xing Tan, Yu Tian, Tongtong Zhang, Qi Yao, Tingting Zhu, Wei Wang, Quanjie Wang, Haiying Fu

**Affiliations:** 1 Fujian University of Traditional Chinese Medicine, Fuzhou, China; 2 Department of Hematology, Rudong People’s Hospital and Affiliated Rudong Hospital of Xinglin College, Nantong University, Nantong, China; 3 Rudong Hospital of Traditional Chinese Medicine, Nantong, China; 4 Department of gynecology, Fuzhou Second General Hospital, Fuzhou, China; 5 Department of Hematology, The Third Affiliated Hospital of Fujian University of Traditional Chinese Medicine, Fuzhou, China

**Keywords:** female, gastrointestinal adverse events, iron deficiency anemia, medication adherence, oral iron preparations

## Abstract

**Background:**

The relationship between oral iron preparation-related gastrointestinal adverse events (AEs) and medication adherence in female patients with iron deficiency anemia (IDA) remains unclear.

**Objective:**

To assess gastrointestinal AEs linked to oral iron preparations via the US FDA Adverse Event Reporting System (FAERS), evaluate medication adherence levels in female IDA patients, and analyze the impact of gastrointestinal AEs and other factors on their medication adherence.

**Methods:**

Reporting odds ratio (ROR) and Proportional Reporting Ratio (PRR) were used to analyze oral iron preparation-related gastrointestinal AEs. The Medication Adherence Report Scale-5 (MARS-5) was used to assess patients’ medication adherence, while logistic regression analyzed the impact of factors (gastrointestinal AEs, medication beliefs, illness perception, doctor-patient relationship, social support) on medication adherence.

**Results:**

FAERS recorded 2,624 reports of gastrointestinal AEs following oral iron supplementation in female patients with IDA (January 2000–March 2025). Disproportionality analysis indicated that ferrous fumarate exhibited the weakest gastrointestinal AE disproportional reporting signal (ROR = 0.36, 95% CI 0.27–0.48; PRR = 0.44), with no positive signals detected, whereas ferrous gluconate displayed the strongest signal (ROR = 2.62, 95% CI 2.05–3.34; PRR = 1.90) and the most prominent positive signals. No significant gastrointestinal AE disproportional reporting signals were found for ferrous sulfate or iron polysaccharide complex. A cross-sectional study (148 patients) showed that adherent patients and non-adherent patients accounted for 64.86% and 35.14%, respectively. Multivariate logistic regression analysis indicated that gastrointestinal AEs were not significantly associated with medication adherence, while medication concern beliefs, doctor-patient relationship, and hemoglobin level were important factors affecting medication adherence in female IDA patients.

**Conclusion:**

Gastrointestinal AEs are not a key factor affecting medication adherence. Reducing patients’ medication concerns, improving the doctor-patient relationship and reinforcing counseling to avoid premature medication withdrawal due to elevated hemoglobin levels are beneficial to enhancing medication adherence in female IDA patients.

## 1 Introduction

Iron deficiency anemia (IDA), one of the most common forms of anemia and nutritional deficiency, has emerged as a critical global public health concern—particularly among women (Stoffel et al., 2020). Owing to their unique physiological characteristics, women are susceptible to IDA due to factors such as heavy menstrual bleeding, pregnancy, and the postpartum period (Cappellini et al., 2022). Thus, effective prevention, screening, and management of IDA are of particular importance. IDA imposes a substantial disease burden. Prior studies indicate that in Asia, the prevalence of IDA among non-pregnant women, pregnant women, and women of childbearing age is 32.5%, 40.1%, and 32.8% respectively (Goodarzi et al., 2020). IDA not only impairs women’s physical work capacity but also hinders neuronal development, negatively affecting cognitive function and memory (Kumar et al., 2022). For pregnant women, its impact can be devastating (Igbinosa et al., 2022). Oral iron supplementation remains the well-established first-line treatment for IDA. However, oral iron preparations are highly prone to causing gastrointestinal adverse reactions resulting in reduced medication adherence in patients and even discontinuing treatment due to intolerance (Ebea-Ugwuanyi et al., 2024).

Medication adherence refers to the degree to which patients correctly administer medications in accordance with physicians’ instructions and treatment regimens (Brown et al., 2016). Factors influencing patients’ medication adherence are multifaceted, encompassing personal beliefs, family dynamics, and interactions with healthcare providers (Mcquaid and Landier, 2018). Previous studies have shown that 40.6% of patients with IDA fail to take their medications regularly or within the recommended timeframe, with gastrointestinal side effects being a key contributing factor (Gereklioglu et al., 2016). Third-generation iron preparations have been widely adopted in clinical practice, demonstrating excellent therapeutic efficacy (Martinez and Leal, 2020; Lu et al., 2025). These agents induce minimal gastrointestinal reactions and are highly acceptable to patients. However, in clinical practice, we still observe some female patients seeking re-treatment due to decreased hemoglobin levels. Beyond specific disease-related factors (i.e., causes of anemia) contributing to reduced hemoglobin, the reasons behind these patients’ self-discontinuation or dosage reduction remain unclear.

Our study aimed to assess gastrointestinal adverse events (AEs) linked to oral iron preparations via the US FDA Adverse Event Reporting System (FAERS), evaluate medication adherence levels in female IDA patients, and analyze the impact of gastrointestinal AEs and other factors on their medication adherence.

## 2 Methods

### 2.1 Association between oral iron preparations and gastrointestinal AEs

Using OpenVigil 2.1, we retrieved data on gastrointestinal AEs associated with oral iron preparations from the FAERS database, covering the period from the first quarter of 2000 to the first quarter of 2025. In this study, the following drugs were selected for investigation based on the clinical medication practice of our hospital: ferrous sulfate, ferrous fumarate, ferrous gluconate, and iron polysaccharide complex. In accordance with MedDRA terminology, we searched for gastrointestinal-related AEs, including nausea, vomiting, diarrhea, abdominal pain, abdominal distension, and others. Additionally, to evaluate the safety of different iron preparations in female patients with IDA, we excluded male IDA patients and non-IDA patients. After deduplication based on information such as ISR number, adverse event, drug, and reporting country, the remaining reports were used for data analysis.

### 2.2 Medication adherence

This study enrolled newly diagnosed female patients with IDA who attended the Hematology Outpatient Department of Rudong County People’s Hospital from June 2020 to June 2025. Inpatients received short-term intravenous iron preparations during hospitalization and switched to oral iron polysaccharide complex for treatment after discharge, whereas outpatients were treated with iron polysaccharide complex. This study provided the following medication guidance to the patients: 1) Regular monitoring of blood routine tests is required (at least once every 2 weeks); 2) The course of oral iron supplementation is about 3–6 months, with the specific duration depending on ferritin test results; 3) Administer the medication during or after meals. Do not take it concurrently with tea or coffee; 4) Black stools may occur during treatment. This is not a cause for concern and does not affect continued medication use. The exclusion criteria for this study are as follows: 1) Patients who are unwilling to participate or unable to cooperate in completing the study; 2) Pregnant women and elderly patients (aged over 65 years). This study was approved by the Ethics Committee of Rudong People’s Hospital. Throughout the research process, we strictly adhered to the relevant requirements of the Declaration of Helsinki.

All patients completed a structured questionnaire 2 months after their initial visit to collect the following information: demographic data (including age, BMI, residential environment, and educational background, et al.), medication adherence, gastrointestinal symptoms, medication beliefs, disease awareness, doctor-patient relationship, social support, and blood routine test results from the previous 2 weeks. The questionnaire was filled out independently by the patients.

In this study, the Medication Adherence Report Scale-5 (MARS-5) was used to assess the medication adherence of patients with IDA who were taking oral iron preparations (Norberg et al., 2022). The MARS-5 questionnaire was scored using a 5-point Likert scale (1 = Always, 5 = Never). Patients with a total score ≥23 were classified as adherent, while those with a total score ≤22 were classified as non-adherent.

We used the Beliefs about Medicines Questionnaire-Specific (BMQ-Specific), the Brief Illness Perception Questionnaire (Brief-IPQ), the Patient-Doctor Relationship Questionnaire (PDRQ-9), and the Perceived Social Support Scale (PSSS) to assess patients’ beliefs about oral iron preparations, patients’ perceptions of IDA, doctor-patient relationship, and social support level, respectively (Hayward et al., 2017; Anh et al., 2025; Wang et al., 2023; Liu et al., 2021).

### 2.3 Statistical analysis

We adopted the Reporting Odds Ratio (ROR) and Proportional Reporting Ratio (PRR) to investigate the potential disproportionate reporting signals association between different oral iron preparations and gastrointestinal AEs (Xiong et al., 2023). A positive signal must meet the following criteria simultaneously: the number of reports on gastrointestinal adverse events is no less than 3, and the lower limit of the 95% Confidence Interval (CI) of ROR exceeds 1. For measurement data conforming to normal distribution, independent samples t-test was used; for measurement data not conforming to normal distribution, rank sum test was used. Chi-square test (χ^2^ test) was used for enumeration data. Binary Logistic regression was used to identify the factors influencing medication adherence in female patients with IDA (the regression variable selection method was the enter method). Indicators with statistical significance in univariate analysis were included in multivariate analysis. To analyze whether the sample size is sufficient to detect the effect of gastrointestinal AE on medication adherence, we conducted a power analysis using the formula: $Power=P\left(Z\;>\;z_{\alpha /2\;}‐\;\sqrt{\frac{N\;\cdot \;{f\;}^{2}}{1+\frac{1\;‐\;\pi }{\pi }\;\cdot \;{f\;}^{2}}}\right)$ , where the f-value (Cohen’s f) was converted from the OR value via the formula $Cohen^{\prime }s\;f=\sqrt{\frac{\ln^{2}\left(OR\right)}{4\pi^{2}/3}}$ (Demidenko, 2007). *P* < 0.05 was considered statistically significant.

## 3 Results

### 3.1 Descriptive analysis

From January 2000 to March 2025, a total of 2,624 reports of gastrointestinal AEs in female patients with IDA following oral iron supplementation were documented. It should be noted that FAERS data only represent reported events and cannot be used to calculate the absolute incidence of adverse events or establish a causal relationship between interventions and events. Among these cases, 1725 (65.74%) were associated with ferrous sulfate, 472 (17.99%) with ferrous fumarate, 311 (11.85%) with ferrous gluconate, and 144 (5.49%) with iron polysaccharide complex. Female IDA patients aged 18–44 years accounted for the largest number of gastrointestinal AEs reports. North America was the region with the highest number of reports, totaling 1,256 (47.87%) cases. The most frequently reported gastrointestinal AE was dyspepsia/acid reflux for both ferrous sulfate (10.26%) and ferrous fumarate (4.24%). In contrast, the most common gastrointestinal AE associated with ferrous gluconate was abdominal pain (40.19%), while for iron polysaccharide complex, it was diarrhea (13.19%, Table 1).

**TABLE 1:** Characteristics of gastrointestinal adverse event reports related to oral iron supplementation.

Characteristic	Ferrous sulfate (n = 1725)	Ferrous fumarate (n = 472)	Ferrous gluconate (n = 311)	Iron polysaccharide complex (n = 144)
Age (years)				
0–17	129 (7.48%)	19 (4.03%)	9 (2.89%)	7 (4.86%)
18–44	459 (26.61%)	90 (19.07%)	38 (12.22%)	61 (42.36%)
45–64	250 (14.49%)	123 (26.06%)	110 (35.37%)	11 (7.64%)
65–74	168 (9.47%)	57 (12.08%)	6 (1.93%)	26 (18.06%)
>75	379 (21.97%)	122 (25.85%)	124 (39.87%)	5 (3.47%)
Unknown	340 (19.71%)	61 (12.92%)	24 (7.72%)	34 (23.61%)
Reporting region				
North America	746 (43.25%)	268 (56.78%)	246 (79.10%)	12 (8.33%)
Europe	450 (26.09%)	123 (26.06%)	32 (10.29%)	3 (2.08%)
Others	251 (14.55%)	49 (10.38%)	17 (5.47%)	127 (88.19%)
Unknown	278 (16.12%)	32 (6.78%)	16 (5.14%)	2 (1.39%)
Outcomes				
Serious outcome	1607 (93.16%)	436 (92.37%)	299 (96.14%)	140 (97.22%)
Death	172 (9.97%)	22 (4.66%)	9 (2.89%)	3 (2.08%)
Life-threatening	100 (5.80%)	38 (8.05%)	24 (7.72%)	3 (2.08%)
Hospitalization	819 (47.48%)	224 (47.46%)	170 (54.66%)	80 (55.56%)
Disability	24 (1.39%)	71 (15.04%)	0	9 (6.25%)
Gastrointestinal AEs				
Nausea	105 (6.09%)	5 (1.06%)	18 (5.79%)	13 (9.03%)
Vomiting	90 (5.22%)	6 (1.27%)	107 (34.41%)	17 (11.81%)
Diarrhea	81 (4.70%)	8 (1.69%)	97 (31.19%)	19 (13.19%)
Constipation	64 (3.71%)	13 (2.75%)	2 (0.64%)	1 (0.69%)
Abdominal pain	59 (3.42%)	9 (1.91%)	125 (40.19%)	7 (4.86%)
Abdominal distension	27 (1.57%)	7 (1.48%)	2 (0.64%)	4 (2.78%)
Dyspepsia/Acid reflux	177 (10.26%)	20 (4.24%)	0	1 (0.69%)
Gastrointestinal bleeding	69 (4.00%)	7 (1.48%)	6 (1.93%)	4 (2.78%)

### 3.2 Disproportionality analysis

Ferrous sulfate was noted to have the highest total number of gastrointestinal AE reports among all preparations; however, no disproportionality signal was identified for overall gastrointestinal AEs with ferrous sulfate use (ROR = 1.12, 95%CI 0.93–1.34; PRR = 1.09; Table 2). This finding indicates that the high reporting volume of ferrous sulfate may be primarily attributable to its high frequency of clinical prescription, rather than reflecting an inherently higher overall gastrointestinal AE reporting disproportionality compared with other iron formulations. In contrast, ferrous fumarate exhibited the weakest gastrointestinal AE disproportionality signal (ROR = 0.36, 95%CI 0.27–0.48; PRR = 0.44), and ferrous gluconate presented the strongest gastrointestinal AE disproportionality signal (ROR = 2.62, 95%CI 2.05–3.34; PRR = 1.90). For iron polysaccharide complex, no significant disproportionality signal for overall gastrointestinal AEs was observed (ROR = 1.04, 95%CI 0.71–1.53; PRR = 1.03).

**TABLE 2:** Signal detection of gastrointestinal adverse events related to oral iron supplementation.

Oral iron preparations	The report number	ROR (*95% CI*)	PRR (*χ* ^*2*^)
All	2624	0.34 (0.01–17.37)	0.51 (0.31)
Ferrous sulfate	1725	1.12 (0.93–1.34)	1.09 (1.33)
Ferrous fumarate	472	0.36 (0.27–0.48)	0.44 (51.92)
Ferrous gluconate	311	2.62 (2.05–3.34)	1.90 (62.98)
Iron polysaccharide complex	144	1.04 (0.71–1.53)	1.03 (0.05)

*ROR* reporting odds ratio, *PRR* proportional reporting ratio, *χ* ^2^ chi-square (chi-square statistics are used to test the statistical significance of the corresponding PRR values).

As shown in Table 3, according to the criteria for positive signals: 1) The positive signals for ferrous sulfate cover nausea, constipation, dyspepsia/acid reflux, and gastrointestinal bleeding. Notably, only dyspepsia/acid reflux showed an extremely strong positive signal (ROR = 14.57, 95%CI 6.82–31.15; PRR = 13.18), warranting targeted clinical vigilance; the remaining signals were mild and non-dominant, which further supports that ferrous sulfate does not exhibit a broadly elevated gastrointestinal AE disproportional reporting despite its high reporting volume. 2) Ferrous gluconate displayed positive signals for vomiting, diarrhea, and abdominal pain, with the strongest positive disproportional reporting signal intensity across all four preparations—this finding solidified its status as the iron formulation with the most pronounced gastrointestinal AE disproportional reporting characteristics. 3) The positive signals for iron polysaccharide complex are limited to nausea and diarrhea, and they are weak positive disproportional reporting signals. This indicates that iron polysaccharide complex is associated with relatively weak gastrointestinal AE disproportional reporting in the FAERS dataset. 4) Ferrous fumarate yielded no positive signals for any gastrointestinal AEs in our analysis, which confirmed that it has the most favorable gastrointestinal AE disproportional reporting profile among the four oral iron preparations evaluated.

**TABLE 3:** Positive signals of gastrointestinal adverse events associated with different oral iron preparations.

Gastrointestinal AEs	Oral iron preparations	The report number	ROR (*95% CI*)	PRR (*χ* ^2^)
Nausea	Ferrous sulfate	105	1.60 (1.08–2.37)	1.56 (5.63)
	Iron polysaccharide complex	13	1.84 (1.01–3.34)	1.76 (4.11)
Vomiting	Ferrous gluconate	107	10.21 (7.56–13.80)	7.04 (311.00)
Diarrhea	Ferrous gluconate	97	9.35 (6.86–12.72)	6.74 (269.79)
	Iron polysaccharide complex	19	1.89 (1.14–3.13)	1.77 (6.24)
Constipation	Ferrous sulfate	64	2.13 (1.22–3.70)	2.08 (7.45)
Abdominal pain	Ferrous gluconate	125	20.33 (14.71–28.12)	12.56 (535.29)
Dyspepsia/Acid reflux	Ferrous sulfate	177	14.57 (6.82–31.15)	13.18 (81.49)
Gastrointestinal bleeding	Ferrous sulfate	69	2.16 (1.26–3.70)	2.12 (8.29)

*ROR* reporting odds ratio, *PRR* proportional reporting ratio, *χ* ^*2*^ chi-square (chi-square statistics are used to test the statistical significance of the corresponding PRR values).

Collectively, ferrous sulfate’s high reporting volume is a reflection of its clinical ubiquity, not higher inherent gastrointestinal AE disproportional reporting relative to other iron formulations; conversely, ferrous fumarate and iron polysaccharide complex demonstrate more favorable gastrointestinal AE disproportional reporting profiles, while ferrous gluconate presents the strongest gastrointestinal AE disproportional reporting signals.

### 3.3 Medication adherence in female patients with IDA

A total of 148 patients were included in this study, of which 96 (64.86%) were classified as adherent patients by the MARS-5, and 52 (35.14%) were classified as non-adherent patients. As shown in Table 4: compared with the non-adherent group, the patients in the adherence group had significantly higher education level (*P* = 0.009), monthly income (*P* < 0.001), and PDRQ scores (*P* < 0.001), while their hemoglobin level within 2 weeks (*P* < 0.001) was significantly lower. In the adherence group, both the patients’ concern beliefs (*P* < 0.001) and the “necessity-concern” difference (*P* < 0.001) were significantly lower than those in the non-adherence group. This indicates that although patients participating in this study generally recognized the value and benefits of iron preparations, the patients in the non-adherence group had concerns about iron preparations at the same time.

**TABLE 4:** Demographic information of the patient.

Variable	Adherent group (n = 96)	Non-adherence group (n = 52)	*t/χ* ^*2*^	*P*
Age (year)	44.80 ± 9.47	45.13 ± 8.39	−0.212	0.832
Body Mass index (kg/m^2^)	23.65 ± 3.54	22.92 ± 3.10	1.248	0.214
Menopause	​	​	0.189	0.710
Yes	28 (29.17%)	17 (32.69%)	​	​
No	68 (70.83%)	35 (67.31%)	​	​
Education level [n (%)]	​	​	7.321	0.009
High school and above	61 (63.54%)	21 (40.38%)	​	​
Junior high school and below	35 (36.46%)	31 (59.62%)	​	​
Marital status [n (%)]	​	​	0.308	0.597
Married	86 (89.58%)	45 (86.54%)	​	​
Unmarried/Divorced	10 (10.42%)	7 (13.46%)	​	​
Monthly income [n (%)]	​	​	15.060	<0.001
<5000 yuan	40 (41.67%)	39 (75%)	​	​
≥5000 yuan	56 (58.33%)	13 (25%)	​	​
Living environment [n (%)]	​	​	0.427	0.605
Urban	50 (10.42%)	30	​	​
Rural	46 (89.58%)	22	​	​
Hypertension [n (%)]	​	​	0.779	0.433
Yes	10 (10.42%)	8 (15.38%)	​	​
No	86 (89.58%)	44 (86.62%)	​	​
Diabetes [n (%)]	​	​	4.236	0.052
Yes	4 (4.17%)	7 (13.46%)	​	​
No	92 (95.83%)	45 (86.54%)	​	​
Smoking [n (%)]	​	​	0.555	0.518
Yes	6 (6.25%)	5 (9.62%)	​	​
No	90 (93.75%)	47 (90.38%)	​	​
Drinking [n (%)]	​	​	0.463	0.624
Yes	15 (15.63%)	6 (11.54%)	​	​
No	81 (84.38%)	46 (88.46%)	​	​
Gastrointestinal symptoms [n (%)]	​	​	5.897	0.023
Yes	2 (2.08%)	6 (11.54%)	​	​
No	94 (2.08%)	46 (88.46%)	​	​
Hemoglobin within 2 Weeks (g/L)	99.67 ± 8.48	108.88 ± 9.91	−5.946	<0.001
BMQ-specific				
Necessity	16.66 ± 2.30	16.71 ± 2.42	0.137	0.891
Concerns	6.64 ± 1.75	12.65 ± 2.76	16.220	<0.001
Necessity - concerns differential	10.02 ± 3.09	4.06 ± 3.81	−10.342	<0.001
PSSS (score)	51.59 ± 7.65	53.52 ± 7.41	−1.478	0.142
PDRQ-9 (score)	19.88 ± 4.15	13.19 ± 2.28	10.746	<0.001

BMQ-specific: Brief illness perception questionnaire, PSSS: Perceived social support scale, PDRQ-9: Patient-doctor relationship questionnaire.

A total of 8 patients (5.41%) developed gastrointestinal AEs in this study. Among them, 2 patients in the adherence group developed constipation after medication; in the non-adherence group, 3 patients reported mild nausea, 2 reported diarrhea, and 1 reported constipation (*P* = 0.023). None of the patients reported vomiting, burning sensation, or abdominal pain.

### 3.4 The patients’ cognition of IDA

Compared with patients in the adherence group, those in the non-adherence group perceived a shorter duration of the disease (*P* < 0.001), paid less attention to the disease (P < 0.001), and had less emotional impact from the disease (*P* < 0.001, Table 5).

**TABLE 5:** The patients’ cognition of IDA: a comparison between the adherence group and the non-adherence group.

Brief-IPQ	Adherence group (n = 96)	Non-adherence Group (n = 52)	*t*	*P*
1. Consequences: How much does your disease (IDA) affect your life?	3.97 ± 1.00	3.83 ± 1.13	−0.758	0.450
2. Timeline: How long do you think the disease (IDA) will last?	4.39 ± 1.02	3.60 ± 0.93	−4.627	<0.001
3. Personal control: To what degree do you believe you are can control your condition (IDA)?	2.81 ± 0.87	2.60 ± 0.85	−1.453	0.148
4.Treatment control: To what extent do you perceive your treatment as beneficial for your disease (IDA)?	3.46 ± 0.96	3.67 ± 0.81	1.368	0.173
5. Identity: To what extent do you think your symptoms stem from your disease (IDA)?	6.36 ± 1.05	6.06 ± 0.92	−1.776	0.078
6. Concern: To what extent are you concerned about your disease (IDA)?	6.08 ± 1.14	5.02 ± 1.02	−5.642	<0.001
7. Coherence: To what degree do you perceive your understanding of your disease (IDA) to be?	5.06 ± 0.84	5.31 ± 1.11	1.505	0.134
8. Emotional representation: To what extent does your disease (IDA) affect your emotions, such as anger, irritability, fear, and depression?	5.58 ± 0.91	4.50 ± 0.94	−6.818	<0.001
Total scores	37.72 ± 2.97	34.54 ± 2.59	−6.502	<0.001

### 3.5 Multivariate logistic regression analysis

We included demographic characteristics, gastrointestinal symptoms, medication beliefs, illness perception, doctor-patient relationship, social support, and blood routine results within 2 weeks in the logistic regression analysis. Results of the univariate analysis showed that gastrointestinal AEs (*OR* = 0.163; *P* = 0.003), educational level (*OR* = 2.537; *P* = 0.007), monthly income (*OR* = 4.200; *P* < 0.001), concern beliefs (*OR* = 2.680; *P* < 0.001), doctor-patient relationship (*OR* = 0.477; *P* < 0.001), illness perception (*OR* = 0.669; *P* < 0.001), and current hemoglobin level (*OR* = 1.120; *P* < 0.001) may be factors affecting medication adherence in patients with IDA. The final multivariate analysis (Table 6) indicated that gastrointestinal AEs were not significantly associated with medication adherence—notably, the achieved power of this study is 0.9989 (far higher than the clinical research threshold of ≥0.80, indicating that the 148-patient sample size is fully adequate to detect the impact of gastrointestinal AEs on medication adherence in female patients with IDA)—while high concern beliefs (*OR* = 2.849; *P* = 0.001), poor doctor-patient relationship (*OR* = 0.652; *P* = 0.019), and high current hemoglobin level (*OR* = 1.222; *P* = 0.011) were factors contributing to poor medication adherence in female patients with IDA.

**TABLE 6:** Multivariate logistic regression analysis of poor medication compliance in patients with IDA.

Variable	*B*	*Walds*	*P*	*OR*	95%*CI*	
					*Upper bound*	*Lower bound*
Gastrointestinal symptoms (no)	−3.045	2.710	0.100	0.048	0.001	1.787
Education level (high school and below)	0.090	0.005	0.942	1.094	0.099	12.037
Monthly income (<5,000 yuan)	−0.213	0.032	0.859	0.808	0.077	8.454
BMQ-specific: Concerns	1.047	10.790	0.001	2.849	1.525	5.321
PDRQ-9 scores	−0.427	5.543	0.019	0.652	0.457	0.931
Brief-IPQ scores	−0.180	0.759	0.384	0.836	0.558	1.428
Hemoglobin level within 2 weeks (g/L)	0.201	6.397	0.011	1.222	1.048	1.428

BMQ-specific: Brief illness perception questionnaire, PSSS: Perceived social support scale, PDRQ-9: Patient-doctor relationship questionnaire.

## 4 Discussion

### 4.1 Association between gastrointestinal AEs and oral iron preparations

In this study, disproportionality analysis was employed to conduct an in-depth investigation into the gastrointestinal AE disproportionality signals linked to four oral iron preparations. Although ferrous sulfate showed no statistically significant disproportionality signal for overall gastrointestinal AEs (ROR = 1.12, 95%CI 0.93–1.34; PRR = 1.09), it had the highest number of reported cases. As the most commonly used oral iron preparation in clinical practice (Santiago, 2012; Pantopoulos, 2024), the high volume of gastrointestinal AE reports for ferrous sulfate may be attributable to its widespread clinical application, rather than reflecting an inherently higher level of gastrointestinal AE reporting disproportionality; this is further supported by the absence of a significantly elevated overall gastrointestinal AE disproportionality signal in our disproportionality analysis. We still need to pay attention to the positive signals exhibited by ferrous sulfate in terms of nausea, constipation, dyspepsia/acid reflux, and gastrointestinal bleeding. Studies have shown that ferrous sulfate, as an inorganic iron salt, is more likely to cause gastrointestinal irritation than organic iron salts (Milman et al., 2014). The potential mechanism behind this phenomenon lies in the fact that Fe^2+^, compared with Fe^3+^, is usually released rapidly and directly in the stomach. High levels of free iron can disrupt the integrity of the gastric mucosal barrier, triggering local inflammatory responses such as mucosal hyperemia and edema, which in turn lead to gastrointestinal reactions (Qi et al., 2020; Pantopoulos, 2024). Additionally, studies have found that excessive Fe^2+^ can penetrate the mucosal defense system and activate enterochromaffin cells to secrete serotonin, thereby inducing nausea and vomiting (Machida and Iizuka, 2023). Our study found that ferrous gluconate was the iron preparation with the strongest gastrointestinal AE disproportional reporting signals, showing strong such signals for vomiting (ROR = 10.21), diarrhea (ROR = 9.35), and abdominal pain (ROR = 20.33). This further confirms the aforementioned characteristics of Fe^2+^. However, surprisingly, ferrous fumarate—another Fe^2+^ preparation—seems to break the above pattern. Ferrous fumarate exhibited excellent performance in terms of gastrointestinal AEs with the weakest disproportional reporting signal for overall gastrointestinal AEs (ROR = 0.36, 95%CI 0.27–0.48; PRR = 0.44) and no positive disproportional reporting signals were detected for any individual gastrointestinal symptoms, thus demonstrating the most favorable gastrointestinal AE disproportional reporting profile overall. Of course, this may also be related to the small sample size. A clinical study showed that ferrous fumarate caused minimal gastrointestinal effects (Milman and Bergholt, 2024), which is consistent with the ROR value (0.36) for overall AEs in our study. Beyond IDA, ferrous fumarate has also demonstrated good tolerability in the treatment of anemia following non-variceal upper gastrointestinal bleeding (Chang et al., 2023). Iron polysaccharide complex showed no significant disproportional reporting signal for overall gastrointestinal AEs (ROR = 1.04, 95%CI 0.71–1.53; PRR = 1.03), with only weak positive signals observed for nausea and diarrhea, indicating a relatively weak overall gastrointestinal AE disproportional reporting profile. We found that among 148 patients treated with iron polysaccharide complex, only 5.41% experienced gastrointestinal reactions, and this, along with multiple studies (Lu et al., 2021; Zhang et al., 2024), confirms the favorable gastrointestinal tolerability of iron polysaccharide complex. Owing to its macromolecular structure, iron polysaccharide complex delivers iron in the form of a stable chelated complex. This chelation mechanism prevents the dissociation of free Fe^2+^, thereby avoiding mucosal irritation (Lo et al., 2023). Furthermore, the exclusive use of iron polysaccharide complex in our clinical cohort may introduce potential bias, which further weakens the link between gastrointestinal AEs disproportional reporting and medication adherence.

### 4.2 The level of medication adherence in patients

In this study, a cross-sectional design was adopted to assess the level of medication adherence in female patients with IDA. Our study results showed that among the included study participants, 96 patients (64.86%) exhibited adherence, while 52 (35.14%) patients were classified as non-adherent. A study conducted in Pakistan reported that without interventions such as providing informational leaflets or physician-led education, 75.54% of patients demonstrated medication adherence at or below the moderate level (Ambreen et al., 2025). Our data are superior to theirs, and this may be associated with the heavier absolute burden of medication costs and relatively poor access to healthcare resources faced by patients living in underdeveloped regions—given that these factors are prone to causing disruptions in healthcare services (Nielsen et al., 2017; Alnajjar et al., 2025).

### 4.3 The factors influencing medication adherence

In this study, binary logistic regression confirmed that concern beliefs were a contributing factor to poor medication adherence in female IDA patients. The cultural roots underlying the relationship between medication adherence and medication concern beliefs can, to a certain extent, explain female IDA patients’ concerns about oral iron preparations (Rees et al., 2014; Belitsi et al., 2023). The deeply ingrained traditional belief in Chinese culture that “all medications carry some degree of toxicity” is a distinctive cultural-specific confounder in the Chinese population, which essentially reflects the public’s dialectical view of medications’ “therapeutic value” and “potential risks.” However, in the context of IDA treatment, this culturally rooted perception may amplify patients’ concerns about the side effects of iron preparations, thereby negatively affecting medication adherence. Notably, this cultural belief is unique to the Chinese context, and the findings pertaining to its impact on medication adherence may have limited generalizability to non-Chinese populations.

Illness perception can usually predict patients’ medication adherence (Smits et al., 2020; Mobini et al., 2023; Matoulkova et al., 2013), and this rule also holds true for patients with IDA. The characteristic of “perceiving a shorter disease duration” in the non-adherent group reflects the cognitive bias of this population regarding the treatment cycle of IDA. As a chronic disease that requires long-term iron supplementation for correction, IDA still necessitates continued iron supplementation to replenish the body’s iron stores even after hemoglobin levels return to normal. However, in clinical practice, patients may misjudge that the disease has been cured and then discontinue medication on their own, often due to symptom relief (e.g., yellowish complexion turning ruddy, persistent improvement or alleviation of fatigue) and recovery of laboratory indicators (hemoglobin approaching or exceeding the lower limit of the normal range). The logistic regression analysis revealed a counter-intuitive yet clinically meaningful finding: patients with higher current hemoglobin levels had poorer medication adherence (OR = 1.222, *P* = 0.011), which strongly corroborates this misjudgment phenomenon. This finding reflects a common cognitive bias among IDA patients—the misconception that normalized or elevated hemoglobin levels equate to complete disease remission, prompting premature and unsupervised medication cessation. Potential reasons for the characteristics of “lower attention to the disease and less emotional impact from the disease” in the non-adherent group may be related to disease-specific features of IDA (e.g., patients with mild anemia may have no obvious symptoms) and the fact that the severity of IDA has a smaller psychological impact on patients compared with diseases such as tumors and cardiovascular diseases. Additionally, a high-quality doctor-patient relationship is a key underpinning for improving patients’ medication adherence (Pena-Valenzuela et al., 2023; Georgopoulou et al., 2020), and our study results also confirm this.

### 4.4 Limitations

Although our study explored the association between oral iron preparations and gastrointestinal AEs by mining the FAERS database, and systematically evaluated the level of medication adherence and its influencing factors in female patients with IDA, several limitations of our study should be noted: 1) As a spontaneous reporting system, the FAERS database has inherent limitations such as high data randomness and missing values for some key variables. This prevents us from further analyzing the impact of important factors such as medication dosage and comorbidities on gastrointestinal AEs. Moreover, FAERS data only represent voluntarily reported adverse events, and thus cannot be used to calculate the absolute incidence of gastrointestinal AEs or directly infer a causal relationship between oral iron preparations and the occurrence of gastrointestinal AEs. 2) There is a wide variety of oral iron preparations in terms of types and dosage forms. The four iron preparations included in this study were selected based on the medication practice of our institution (a primary hospital), which limits their representativeness. Only iron polysaccharide complex was administered in our clinical cohort, and this single-drug design introduces another key limitation: our conclusion that there is no association between gastrointestinal adverse events and medication adherence is only applicable to patients receiving this well-tolerated iron preparation, and cannot be generalized to patient populations treated with more irritant iron formulations (e.g., ferrous sulfate). In addition, the severity of gastrointestinal AEs was not captured in this study. Future studies should incorporate a broader range of oral iron preparations and capture gastrointestinal AE severity in the questionnaire to address these limitations of insufficient generalizability and incomplete gastrointestinal AE-related data collection. 3) The sample size of this cross-sectional study was relatively small, and the conclusions thus drawn require validation by large-sample studies.

## 5 Conclusion

Our findings provide valuable references for rational clinical drug use. Medication adherence in female patients with IDA is relatively poor. Gastrointestinal AEs were not a key factor influencing medication adherence in female IDA patients. Based on the results of logistic regression, minimizing patients’ concerns about medications, further improving the doctor-patient relationship and reinforcing counseling to avoid premature medication withdrawal due to elevated hemoglobin levels are favorable measures to enhance medication adherence in female IDA patients.

## Data Availability

The original contributions presented in the study are included in the article/supplementary material, further inquiries can be directed to the corresponding author.
